# Tail Biting in Pigs: Blood Serotonin and Fearfulness as Pieces of the Puzzle?

**DOI:** 10.1371/journal.pone.0107040

**Published:** 2014-09-04

**Authors:** Winanda W. Ursinus, Cornelis G. Van Reenen, Inonge Reimert, J. Elizabeth Bolhuis

**Affiliations:** 1 Adaptation Physiology Group, Department of Animal Sciences, Wageningen University, Wageningen, The Netherlands; 2 Animal behaviour & Welfare, Wageningen UR Livestock Research, Wageningen, The Netherlands; ETH Zurich, Switzerland

## Abstract

Tail biting in pigs is a widespread problem in intensive pig farming. The tendency to develop this damaging behaviour has been suggested to relate to serotonergic functioning and personality characteristics of pigs. We investigated whether tail biting in pigs can be associated with blood serotonin and with their behavioural and physiological responses to novelty. Pigs (n = 480) were born in conventional farrowing pens and after weaning at four weeks of age they were either housed barren (B) or in straw-enriched (E) pens. Individual pigs were exposed to a back test and novel environment test before weaning, and after weaning to a novel object (i.e. bucket) test in an unfamiliar arena. A Principal Component Analysis on behaviours during the tests and salivary cortisol (novel object test only) revealed five factors for both housing systems, labeled ‘Early life exploration’, ‘Near bucket’, ‘Cortisol’, ‘Vocalizations & standing alert’, and ‘Back test activity’. Blood samples were taken at 8, 9 and 22 weeks of age to determine blood platelet serotonin. In different phases of life, pigs were classified as tail biter/non-tail biter based on tail biting behaviour, and as victim/non-victim based on tail wounds. A combination of both classifications resulted in four pig types: biters, victims, biter/victims, and neutrals. Generally, only in phases of life during which pigs were classified as tail biters, they seemed to have lower blood platelet serotonin storage and higher blood platelet uptake velocities. Victims also seemed to have lower blood serotonin storage. Additionally, in B housing, tail biters seemed to consistently have lower scores of the factor ‘Near bucket’, possibly indicating a higher fearfulness in tail biters. Further research is needed to elucidate the nature of the relationship between peripheral 5-HT, fearfulness and tail biting, and to develop successful strategies and interventions to prevent and reduce tail biting.

## Introduction

Aberrant behaviours such as tail biting, i.e. the harmful oral manipulations of the group mates’ tails, can both reflect and contribute to health and welfare problems in pigs [1]. Tail biting is caused by many factors [2], but the lack of exploration possibilities in the home pen is likely the main risk factor for its development [3]. Accordingly, tail biting is generally seen as redirected explorative behaviour [4]. Indeed, pigs kept in pens enriched with materials suitable for chewing and rooting, perform much less tail biting compared to pigs kept in rather barren pens [3], [5]. However, in barren housing systems not all pigs develop tail biting behaviour, and, conversely, in enriched housing systems still some pigs perform the behaviour [6]. Previously, the existence of different types of tail biters was suggested [2] and there may be different underlying motivations to display the damaging behaviour by pigs kept in diverging housing systems. Individual pigs may therefore be predisposed to develop tail biting behaviour. Tail biting behaviour has comparable characteristics with for instance feather pecking in laying hens [7] and feather picking in parrots [8]. The behaviours involved may be perceived as maladaptive as they seem an inadequate response given the living circumstances [9], [10]. However, an important commonality of the problematic behaviours may be a malfunctional neurotransmitter system [11], [12] which would imply that in some individuals the behaviours may be malfunctional rather than maladaptive only [10]. Furthermore, it has been suggested that the propensity to develop aberrant behaviour is related to personality traits, such as the strategy (or ‘coping style’ [13]) an animal adopts in challenging situations [11], [14]. Animals may be classified in either proactive or reactive individuals [13]. Briefly, proactive animals cope more actively with acute mild stress, develop more easily routines and seem more rigid in their responses to changes in their environment, whereas reactive animals respond more passively to acute stressors and seem more flexible in their behavioural responses [15], [16]. These coping styles may also relate to the functioning of the brain neurotransmitter systems [17], [18] and it was previously suggested that these proactive animals may be more vulnerable to develop compulsive disorders, which show similarities with feather pecking in laying hens and tail biting in pigs [11]. Another personality trait that may contribute to the tendency of animals to develop aberrant behaviours is fearfulness or anxiety [19], [20] which may be reflected in the response to novel situations [21], [22]. Additionally, in pigs the behaviour in novelty tests has been associated with coping styles [23], tail biting [20], and serotonergic blood and brain parameters [22]. Therefore, the main aim of our study was to explore whether tail biting in pigs, in a longitudinal study, is associated with behavioural and physiological responses to challenges, and also with blood serotonin. Blood serotonin is relatively easy to measure compared to brain serotonin and behaviours and may, thus, be valuable in understanding the mechanisms of tail biting. Pigs were subjected to a back test as the behaviour performed during this test may reflect a pig’s coping style [16], [24], and they were exposed to two novelty tests, one before and one after weaning. After weaning, salivary cortisol measured around the novelty test was used to assess the pig’s adrenal response to a challenging situation [25]. Tail biting behaviour, tail damage, and blood serotonin were measured at different time points in life as we recently found that tail biting in pigs is not always consistent over different life stages [6]. Previously, in laying hens [26] and pigs [27] differences were found in gene expression profiles of the animals that performed damaging behaviours (feather peckers/tail biters), animals that received the damaging behaviours (victims of feather pecking/tail biting), and animals that were not involved in performing or receiving the damaging behaviours (neutral animals). Apart from focussing on pigs displaying tail biting behaviour, it seems, therefore, highly relevant to explore other types of pigs as well. Accordingly, our pigs were classified in tail biters/non tail biters and victims/non-victims of tail biting. Combining both classifications resulted in biters, victims, a combination of both (biter/victims), and pigs that never engaged in tail biting or receiving the behaviour (neutrals). We chose to use both barren and enriched pens as environmental enrichment strongly affects the prevalence of tail biting behaviour (e.g. [6], [28]), and may reveal different types of tail biters [2].

## Materials and Methods

### Ethics Statement

The experimental protocol followed during this study was approved by the Animal Care and Use Committee of Wageningen University (no. 2010055f) and then also adopted by the Animal Care and Use Committee of the University of Groningen, the Netherlands. Blood samples were taken near the home pens of the pigs and as quickly as possible to minimize stress. Pigs with severe tail wounds (i.e. tip of tail missing) were removed from the experiment and all pens (barren and enriched) received a jute sack from 8 weeks of age onward to keep tail biting in barren housing at an acceptable level.

### Animals and housing

Pigs (n = 480) were born in 5 rounds at the experimental farm of TOPIGS Research Center IPG in Beilen (the Netherlands). Briefly, piglets were housed in a conventional (barren) farrowing pen with a sow crate. Tails and teeth were kept intact, but males were castrated. At four weeks of age piglets were transported to the experimental farm “De Haar” in Wageningen (the Netherlands). After weaning pigs were kept either barren (B) or enriched (E). The difference between E and B housing was the provision of wood shavings (12 kg at start, 3 kg added daily) and straw (1.5 kg daily) in E housing. B housed pigs received two handfuls of wood shavings daily, from six weeks of age onward. Additionally, from week 8 onward both B and E pens received a jute sack, to keep tail biting at an acceptable level in B pens. More details on housing of the pigs both pre- and post-weaning have previously been described (see [6], [24], [29]). Groups of pigs in a pen also differed in Indirect Genetic Effects (IGE) for growth; IGE results are presented elsewhere (see [30], [31]). Each pen consisted of six unrelated pigs with a 1∶1 sex ratio, and at least two pigs of each back test (see below) classification (LR:HR ratio [24]).

### Tail biting behaviour and tail damage

Four life phases were distinguished: one pre-weaning, and three post-weaning (1–3). These phases were, roughly, according to general production stages: piglet (0–4 weeks), weaner (4–8 weeks), grower (8–16 weeks), finisher (16–23 weeks). Pigs were, per phase, identified as tail biters and victims of being bitten based on home pen observations and tail damage scores, respectively [6]. Tail biters were pigs involved in more than one tail biting incidence (i.e. nibbling, sucking or chewing at the tail of a pen mate) during a phase post-weaning (>1 out of 360 samples). Pre-weaning, tail biters could not be identified properly and were, therefore, not considered in this study. Tail biting behaviour was observed during instantaneous scan samples with an interval of two min (30 samples/h) while using a Psion Workabout with Observer software (Noldus Information Technology, Wageningen, The Netherlands). In total six observation days (at 4, 5, 8, 11, 16, and 21 weeks of age) of six hours each (in total 1080 samples per pig) were considered and per phase two observation days (i.e. 360 samples per pig) were used. Victims of tail biting were pigs with a tail wound (i.e. any skin damage involving (clotted) blood, which refers to the severest tail damage score recorded, see [6]) at time of weaning, or at least one time during a phase post-weaning (weeks 5–7, 8–15, and 16–23 for phase 1, 2 and 3, respectively) [6].

### Behavioural tests

#### Pre-weaning back test

**Figure 1:**
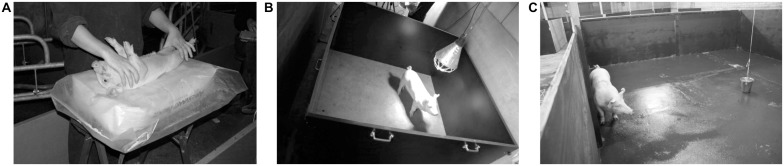
Pigs subjected to three behavioural tests. **A:** Back test. **B:** Pre-weaning novel environment exposure (pNEe). **C:** Novel object exposure (NOe).

Piglets (n = 480) were subjected to a back test at approximately 14 days of age (Figure 1A) (see [24]). The test was carried out on two consecutive days, except for the final round where all 96 piglets were tested in one day. Preliminary analyses showed that time of day did not significantly effect the results. From each litter, individual piglets were placed in supine position for one min to observe their behavioural response which may range from vigorous struggling and screaming to immobility (see for more details [32], [33]). Two observers conducted the test, one observer held the piglet and counted the number of struggles, and the other observer counted the number of vocalizations and registered latency times to first struggle and vocalization. Latency to first struggle was strongly correlated with number of struggles (r = −0.84, P<0.001) and latency to first vocalization was strongly correlated with number of vocalizations (r = −0.76, P<0.001) (analysed with Spearman’s rank correlation on residuals from a GLM with round as fixed effect, n = 480). Therefore, further analysis was restricted to numbers of struggles and vocalizations.

#### Pre-weaning novel environment exposure

Pre-weaning, individual piglets (n = 470) were, by litter, exposed to a novel environment (pNEe) at the age of 3.5 weeks (see also [24]). Testing was, per round, carried out on two consecutive days. Preliminary analyses showed that time of day did not significantly effect the results. The novel environment consisted of a 1.25 m×1.25 m arena with dark wooden walls of 62.5 cm height, and concrete flooring (Figure 1B). A heating lamp with yellow lighting was placed above the arena. The test lasted for 2.5 min. Behaviours included in our study were percentage of time spent on walking (all forms of moving), standing alert (standing with head upward and ears pricked), exploring (walking or standing while nosing, licking or rooting floor or walls), and total number of vocalizations (grunts, grunt-squeals, squeals, and screams). Behaviours were recorded by two observers (one for continuous behaviours and one for behavioural events) who had each a Psion Workabout with Observer software (Noldus Information Technology, Wageningen, The Netherlands).

#### Post-weaning novel object exposure

Post-weaning, individual pigs (n = B: 224, E: 227) were subjected to a novelty test at 13 weeks of age (see also [30]). The order of testing was balanced for housing condition and sex. Testing was, per round, carried out on five consecutive days. The size of the test arena was 5 m×5 m, made of wooden walls (approximately 1.5 m high) and concrete flooring (Figure 1C). The arena (walls + floor) was darkened with grey painting. The first 5 min pigs were exposed to the test arena (i.e. novel environment exposure or NEe), then a metal bucket was dropped from the ceiling (i.e. novel object exposure or NOe) and pigs were left in the arena for another 5 min. Total duration of the test was 10 min. In a previous study large behavioural differences were found between NEe and NOe when pigs were 11 weeks of age, pointing in the direction of NOe being more fearful for pigs post-weaning compared to NEe [22]. Since we were especially interested in behavioural responses to potentially stressful situations, only the behaviours recorded during the NOe were used for further analysis. Behaviours considered were percentage of time spent on standing alert (standing with head upward and ears pricked), exploring the arena (nosing and rooting floor, nosing and rooting walls, and chewing), exploring the novel object (nosing, sniffing, rooting, licking or chewing the metal bucket), and total number of vocalizations (barks, grunts, grunt-squeals, squeals, and screams). Two observers with a Psion hand-held computer recorded continuous behaviours or behavioural events. Additionally, total distance covered (i.e. locomotion), and percentage of time spent near the door (∼1.25×2.25 m in front of the door, with the door centred in the ∼2.25, see also [30]) and directly adjacent to the walls (excluding the door zone) were recorded by video tracking using EthoVision XT 8.5 (Noldus Information Technology, Wageningen, The Netherlands). Housing (B/E) effects on behavioural parameters of the NOe were not present, which was previously described elsewhere [30], and are therefore not further considered in our study.

### Physiological measures

#### Serotonin measures

Blood samples were taken when the pigs were approximately 8 (T1), 9 (T2), and 22 (T3) weeks of age. The first (n = B: 237, E: 235 pigs) and last blood samples (n = B: 213, E: 221 pigs) were considered as basal samples. The second sample (n = B: 233, E: 230 pigs) was taken three days after a regrouping test, where pairs of pigs were, for another study, mixed with unfamiliar pigs during 24 h and then returned to their original home pen (see for more details [29]), but no short-term effects on blood serotonergic measures were expected as they are thought to be rather stable over time [34], [35]. Samples were taken by venipuncture while the pigs were either placed on their backs (T1 and T2) or when held in a nose sling (T3). Order of blood collection was always balanced for housing. Blood was collected in EDTA containing tubes (9 ml) and stored on ice until transfer to the lab for further processing. Platelets (expressed in 10^9^ cells/l) were counted using a Sysmex F-820 Counter (Sysmex Corporation, Kobe, Japan).

*Blood platelet serotonin level*. Platelet serotonin (5-hydroxytryptamine or 5-HT) level was determined using a fluorimetric assay based on [36], [37] as previously described [22]. Platelet 5-HT level was expressed in nmol/10^9^ platelets.

*Whole blood serotonin level.* Serotonin in platelets represents >95% of 5-HT found in blood [38], [39]. Therefore, we multiplied platelet 5-HT level by the number of platelets counted in whole blood (10^9^ cells/l) to obtain whole blood 5-HT. Whole blood 5-HT was expressed in nmol/ml blood.

*Blood platelet serotonin uptake velocity.* Serotonin uptake velocity in blood platelets was measured by a radioactive uptake assay based on [40], [41] as previously described [22]. Platelet 5-HT uptake velocity was expressed as pmol/10^9^ platelets/min.

#### Salivary cortisol measures

Before (t = 0 min) and after (t = 15, 30, and 60 min) the start of the novelty test at 13 weeks of age, pigs (n = B: 218, E: 218) were allowed to chew on two cotton buds to obtain saliva samples to measure cortisol concentrations by a radioimmunoassay kit [30], [42]. Pigs were previously habituated to chewing on the cotton buds. For each pig the deltas between the first and second sample (Δ t = 15–0) (i.e. cortisol rise from the first sample to the highest observed peak), and between the second and final sample (Δ t = 15–60) (i.e. cortisol recovery from the highest observed peak to the final sample) were calculated. The area under the curve (AUC) from time 0 to 60 was determined per pig using the linear trapezoidal rule. Housing (B/E) effects on salivary cortisol parameters were previously described elsewhere [30] and therefore not further considered in our study. Shortly, B housed pigs had consistently higher values compared to E housed pigs, but cortisol responses to novelty did not differ between the pigs from the two different housing types.

### Statistical Analysis

SAS version 9.2 (Statistical Analysis System Institute and Inc., 2002–2008) was used for all statistical analyses. The effect of type of pig (see below) was considered per phase as tail biting behaviour observed in individual pigs was inconsistent throughout life resulting in different classifications per pig per phase [6]. Additionally, an ‘overall’ score per pig (ever (i.e. in one of the three phases) a tail biter, and ever a victim or not) was used to assess the overall effect of type of pig on variables considered per phase. Number of pigs varied among analyses due to several reasons: some pigs did not participate in the novel environment test pre-weaning (i.e. they were initially not selected for follow up), pigs were removed from the experiment due to health reasons and tail wounds, blood samples were not suitable for analysis, some blood and saliva samples were lost or could not be analysed due to technical problems.

#### The effect of housing and of type of pig on serotonin measures

The effect of housing (B/E) on serotonin measures was tested in a mixed model with a fixed effect of round (1–5) and random effect of pen (nested within round). Subsequently, the effect of type of pig with respect to tail biting (per phase and over all phases) on serotonin measures was tested separately for both types of housing (B/E). At time of weaning piglets were classified as either a victim (i.e. piglet had a tail wound; n = B: 14, E: 30) or non-victim of tail biting (i.e. piglet had no tail wound; n = B: 226, E: 210). This classification in non-victim/victim (0/1) was tested as fixed effect in a mixed model, together with round (1–5) and the random effect of pen (nested within round). In each phase (1–3) post-weaning, pigs were classified as either a tail biter (at least twice involved in biting incident) (n = B: 29, 40, and 38, E: 0, 6, and 8 in phase 1–3, respectively) or a non-tail biter (n = B: 211, 199, and 183, E: 240, 232, and 223 in phase 1–3, respectively). In addition, all pigs were classified in each phase as either a victim (at least once a tail wound) (n = B: 76, 132, and 154, E: 26, 29, and 58 in phase 1–3, respectively) or a non-victim (no tail wounds) (n = B: 164, 107 and 68, E: 210, 214, 209 and 173 in phase 1–3, respectively). Both 0/1 classifications were treated as fixed effects with two levels, i.e. the effects of ‘biter’ (non-tail biter = 0 versus tail biter = 1) and of ‘victim’ (non-victim = 0 versus victim = 1), respectively. These two fixed effects and their interaction were included in a mixed model, together with the fixed effect of round (1–5) and the random effect of pen (nested within round). Including the interaction between the effects of ‘biter’ and of ‘victim’ in the model made it possible to distinguish between the following four types of pigs: biter (‘biter’ = 1, ‘victim’ = 0) (n = B: 23, 22, and 11, E: 0, 5, and 2 for phase 1–3, respectively), victim (‘biter’ = 0 and ‘victim’ = 1) (n = B: 70, 114, and 126, E: 26, 28, and 52 for phase 1–3, respectively), a combination of both, here referred to as biter/victim (‘biter’ = 1 and ‘victim’ = 1) (n = B: 6, 18, and 27, E: 0, 1, and 6 for phase 1–3, respectively) and pigs not involved in tail biting or being tail bitten, here referred to as neutral pigs (‘biter’ = 0 and ‘victim’ = 0) (n = B: 141, 85, and 57, E: 214, 204, and 171 for phase 1–3, respectively). A similar approach was used to test the effect of an overall classification of each pig. Pigs were classified as tail biter if they were at least once a tail biter in any of the phases (n = B: 88, E: 14). Similarly, pigs were classified in victims if they were at least once a victim in any of the phase (n = B: 200, E: 109). Including the interaction of the effects of ‘biter’ and ‘victim’ resulted in biters (n = B: 15, E: 4), victims (n = B: 127, E: 99), biter/victims (n = B: 73, E: 10) and neutrals (n = B: 25, E: 127). Serotonin measures were logarithmically transformed if necessary to approach normal distribution of residuals.

#### Principal Component Analysis

A Principal Component Analysis (PCA) [43] was conducted by type of housing (B/E) on variables from the back test, pre-weaning novel environment test (pNEe), and novel object test (NOe) including salivary cortisol measures, to examine whether variation in behavioural and physiological responses of the pigs (17 in total) could be summarized in a limited number of different factors [44]. Prior to PCA, variables were, if necessary, square root (number of vocalizations), arcsine square root (proportion of time spent exploring the bucket, and proportion of time spent near the door zone during NOe) or logarithmically (cortisol measures) transformed, and all variables were subjected to a general linear model with round (1–5) as fixed effect to obtain residuals used for the PCA. After extraction, principal components were scaled by their standard deviations (square roots of associated Eigenvalues) and subjected to orthogonal rotation (varimax) to obtain independent factors.

#### The effect of type of pig on principal component factors

A mixed model was performed to test whether type of pig (over all phases and per phase) with respect to tail biting, had an effect on the factors that were retained from the PCA. Similar to the analysis of serotonin measures, 0/1 classifications of pigs as ‘biter’ (non-tail biter = 0 versus tail biter = 1) and ‘victim’ (non-victim = 0 versus victim = 1) were included in the model as fixed effects, and their interaction was considered as well (resulting in the four pig types: biters, victims, biter/victims, neutrals). Furthermore, the random effect of pen (nested within round) was included in the model. Round (1–5) was not included in the model, as variables were already corrected for the effect of round prior to PCA.

#### Correlations between serotonin measures and principal component factors

To assess consistency over time (T1, T2, and T3) with respect to blood serotonin storage (both expressed in whole blood and in blood platelets), a general linear model per type of housing (B/E) with round (1–5) as fixed effect was run on raw data of serotonin variables to obtain residuals. Thereafter, Spearman’s rank correlation coefficients (as not all variables approached normality) were calculated between residuals of serotonin measures. A similar procedure was used to determine possible relationships between serotonin measures and factors obtained by the PCA. Spearman’s rank correlation coefficients were calculated between residuals of serotonin measures and factors.

## Results

### The effect of housing and of type of pig on serotonin measures

Housing significantly affected platelet 5-HT uptake velocity (determined at T3 only), where B housed pigs had higher velocities compared to E housed pigs (P<0.05) (Table 1). No other significant effects of housing were found with respect to 5-HT measures, except that B housed pigs tended to have higher platelet 5-HT levels at T1 (P<0.10).

**Table 1:** Blood 5-HT measures at 8 (T1), 9 (T2), and 22 (T3) weeks of age in barren or enriched housed pigs^a^.

Blood measures^b^	Barren	Enriched	*P*-value
	*n = 203–237*	*n = 207–235*	
Whole blood 5-HT T1	12.6±0.52	11.4±0.52	
Whole blood 5-HT T2	12.0±0.60	11.9±0.60	
Whole blood 5-HT T3	7.3±0.48	7.6±0.48	
Platelet 5-HT uptake T3	45.4±1.43	40.1±1.41	*
Platelet 5-HT level T1	22.9±0.80	21.3±0.80	+
Platelet 5-HT level T2	20.2±1.00	20.7±1.01	
Platelet 5-HT level T3	18.4±0.91	19.5±0.90	

a Untransformed LSmeans ± SEM.

b Whole blood 5-HT level in nmol/ml; Platelet 5-HT level in nmol/platelet 10^9^; Platelet 5-HT uptake velocity in pmol/platelet 10^9^/min.

+P<0.10.

*P<0.05.

Whole blood 5-HT measured at T1 was significantly (P<0.001) positively correlated with whole blood 5-HT measured at T2 (B: r = 0.42, E: r = 0.44) and T3 (B: r = 0.40, E: r = 0.36), and T2 and T3 were also significantly (P<0.001) correlated (B: r = 0.43, E: r = 0.38). Corresponding results were found for blood platelet 5-HT measures (all P<0.001), where T1 was correlated with T2 (B: r = 0.43, E: r = 0.41) and T3 (B: r = 0.37, E: r = 0.38), and T2 was also correlated with T3 (B: r = 0.37, E: r = 0.29).

#### Barren housing

Considering a pig’s life as a whole, no significant main effects of ‘biter’ (i.e. non-tail biters versus tail biters) or ‘victim’ (i.e. non-victims versus victims), and no significant interaction between these effects (i.e. allowing for the distinction between tail biters, victims, biter/victims, and neutral pigs) were found with respect to the blood 5-HT measures (data not shown). However, considering each phase of life separately did reveal relationships between type of pigs and serotonin measures. Victims at time of weaning had, when B housed post-weaning, lower whole blood and platelet 5-HT levels at T1 and T3 compared to non-victims (Table 2). Tail biters classified during phase 1 post-weaning tended to have a lower platelet 5-HT level at T1 compared to non-tail biters (Table 3). Tail biters of phase 2 had a significantly lower platelet 5-HT level at T2 and they tended to have a lower whole blood 5-HT level at T2 compared to non-tail biters. Furthermore, phase 3 tail biters had significantly lower platelet 5-HT levels at T2 compared to non-tail biters. Victims of phase 3 had significantly lower platelet 5-HT uptake velocities, and tended to have higher platelet 5-HT levels at T2 compared to non-victims. Finally, a significant interaction between ‘biter’ and ‘victim’ revealed that tail biters of phase 3 had lower whole blood 5-HT at T2, and tended to have higher platelet 5-HT uptake velocities at T3 compared to victims, biter/victims, and neutral pigs.

**Table 2:** Blood 5-HT measures at 8 (T1), 9 (T2), and 22 (T3) weeks of age in barren or enriched housed victims and non-victims of tail biting at time of weaning^a^.

Blood measures^b^	Victim	Non-victim	*P*-value
**Barren**	*n = 12–13*	*n = 191–224*	
Whole blood 5-HT T1	8.1±1.75	12.8±0.59	**
Whole blood 5-HT T2	10.0±1.94	12.1±0.61	
Whole blood 5-HT T3	2.1±1.30	7.6±0.49	***
Platelet 5-HT uptake T3	45.6±5.96	45.3±1.48	
Platelet 5-HT level T1	17.7±2.76	23.1±0.91	*
Platelet 5-HT level T2	19.4±3.02	20.3±0.99	
Platelet 5-HT level T3	11.2±2.73	18.9±0.73	**
**Enriched**	*n = 24–29*	*n = 183–206*	
Whole blood 5-HT T1	11.3±1.13	11.4±0.46	
Whole blood 5-HT T2	13.2±1.35	11.7±0.57	
Whole blood 5-HT T3	8.1±1.12	7.5±0.51	
Platelet 5-HT uptake T3	45.5±3.48	39.4±1.52	+
Platelet 5-HT level T1	21.4±1.97	21.3±0.77	
Platelet 5-HT level T2	25.8±2.35	20.0±0.95	
Platelet 5-HT level T3	19.8±2.62	19.5±1.09	

a Untransformed LSmeans ± SEM.

b Whole blood 5-HT level in nmol/ml; Platelet 5-HT level in nmol/platelet 10^9^; Platelet 5-HT uptake velocity in pmol/platelet 10^9^/min.

+P<0.10.

*P<0.05.

**P<0.01.

***P<0.001.

**Table 3:** Blood 5-HT measures at 8 (T1), 9 (T2) and 22 (T3) weeks of life and given per type of pig (with respect to tail biting) in barren housing over three phases of life (weaner, grower, finisher) post-weaning^a^.

					*P*-values^c^		
Blood measures^b^	Biter	Victim	Biter/Victim	Neutral	B	V	B×V
***Phase 1***	*n = 21–23*	*n = 56–67*	*n = 3–6*	*n = 122–141*			
Whole blood 5-HT T1	11.3±1.36	14.6±0.91	11.1±2.57	11.9±0.70			
Whole blood 5-HT T2	12.8±1.58	12.7±0.98	10.9±2.87	11.5±0.73			
Whole blood 5-HT T3	7.0±1.05	7.4±0.73	6.5±2.06	7.3±0.57			
Platelet 5-HT uptake T3	42.9±4.31	49.4±2.81	43.4±11.67	43.7±1.93			
Platelet 5-HT level T1	19.1±2.15	23.9±1.43	18.1±4.06	23.2±1.09	+		
Platelet 5-HT level T2	22.4±2.45	21.3±1.55	18.6±4.44	19.4±1.17			
Platelet 5-HT level T3	16.6±2.04	18.2±1.32	14.6±4.25	19.0±0.90			
***Phase 2***	*n = 19–21*	*n = 98–114*	*n = 16–18*	*n = 70–84*			
Whole blood 5-HT T1	10.3±1.42	12.7±0.73	14.0±1.53	12.6±0.83			
Whole blood 5-HT T2	10.5±1.60	12.0±0.78	10.9±1.72	12.5±0.88	+		
Whole blood 5-HT T3	6.9±1.11	7.2±0.59	6.4±1.21	7.7±0.66			
Platelet 5-HT uptake T3	49.2±4.80	45.1±2.09	46.2±5.18	44.5±2.52			
Platelet 5-HT level T1	20.2±2.23	22.3±1.13	22.4±2.39	24.3±1.27			
Platelet 5-HT level T2	17.9±2.48	20.3±1.24	18.1±2.67	21.0±1.40	*		
Platelet 5-HT level T3	19.6±2.24	18.8±1.00	15.8±2.42	18.2±1.14			
***Phase 3***	*n = 7–11*	*n = 116–124*	*n = 23–27*	*n = 52–57*			
Whole blood 5-HT T1	11.7±1.96	12.7±0.73	11.6±1.28	12.4±0.97			
Whole blood 5-HT T2	7.0±2.15^a^	11.7±0.68^b^	12.3±1.32^b^	11.5±0.96^b^	**	**	*
Whole blood 5-HT T3	5.7±1.42	6.8±0.47	6.60±0.90	7.5±0.65			
Platelet 5-HT uptake T3	63.2±7.66^y^	44.1±1.90^z^	48.6±4.28^z^	44.6±2.85^z^	+	*	+
Platelet 5-HT level T1	25.7±3.12	22.2±1.14	25.4±2.03	22.6±1.53			
Platelet 5-HT level T2	15.9±3.42	19.8±1.10	21.7±2.10	18.8±1.54	*	+	
Platelet 5-HT level T3	21.3±2.89	17.8±0.85	16.8±1.84	18.5±1.23			

a Untransformed LSmeans ± SEM. LSmeans lacking a common letter differ by P<0.05 (a/b) or P<0.10 (y/z).

b Whole blood 5-HT level in nmol/ml; Platelet 5-HT level in nmol/platelet 10^9^; Platelet 5-HT uptake velocity in pmol/platelet 10^9^/min.

c B = Main effect of tail biter; V = Main effect of victim; B×V = Interaction between main effects resulting in Biter, Victim, Biter/Victim and Neutral.

+P<0.10.

*P<0.05.

**P<0.01.

#### Enriched housing

Also when considering E housed pigs throughout life, no relationships were found between type of pigs (in terms of either significant fixed effects of ‘biter’ and ‘victim’ or their interaction) and 5-HT measures, although a tendency for an interaction between tail biters and victims was found with respect to platelet 5-HT measured at T3 (P<0.10, but post-hoc analysis revealed no pairwise differences; untransformed LSmeans ± SEM: biters: 14.4±6.62, victims: 19.3±1.55, biter/victims: 26.7±4.85, neutrals: 19.3±1.38) (non-significant findings not shown). Victims at time of weaning tended to have a higher platelet 5-HT uptake velocity at T3 compared to non-victims when E housed post-weaning (Table 2). Pigs identified as victim of tail biting during phase 2 post-weaning had lower whole blood 5-HT levels and platelet 5-HT levels at T3 compared to non-victims (Table 4). Tail biters identified during phase 2 had higher platelet 5-HT uptake velocities at T3 compared to non-tail biters. In phase 3, a significant interaction was observed between ‘biter’ and ‘victim’, where tail biters tended to have lower whole blood 5-HT levels at T3 compared to biter/victims and neutral pigs. A significant interaction between ‘biter’ and ‘victim’ was also found for platelet 5-HT levels at T3 (P<0.01), where tail biters had significantly lower levels compared to biter/victims and neutral pigs, and tended to have lower levels compared to victims of tail biting.

**Table 4:** Blood 5-HT measures at 8 (T1), 9 (T2) and 22 (T3) weeks of life and given per type of pig (with respect to tail biting) in enriched housing over three phases of life (weaner, grower, finisher) post-weaning^a^.

					*P*-value^c^		
Blood measures^b^	Biter	Victim	Biter/victim	Neutral	B	V	B×V
***Phase 1***	*n = 0*	*n = 22–25*	*n = 0*	*n = 185–210*			
Whole blood 5-HT T1	−	11.7±1.26	−	11.3±0.46	−		−
Whole blood 5-HT T2	−	12.7±1.51	−	11.8±0.57	−		−
Whole blood 5-HT T3	−	10.1±1.28	−	7.3±0.49	−		−
Platelet 5-HT uptake T3	−	41.6±3.87	−	40.0±1.51	−		−
Platelet 5-HT level T1	−	20.8±2.19	−	21.4±0.77	−		−
Platelet 5-HT level T2	−	21.9±2.64	−	20.6±0.95	−		−
Platelet 5-HT level T3	−	27.5±2.95	−	18.6±1.05	−		−
***Phase 2***	*n = 4–6*	*n = 26–27*	*n = 0–1*	*n = 177–202*			
Whole blood 5-HT T1	11.9±2.60	12.0±1.16	12.9±5.83	11.3±0.46			
Whole blood 5-HT T2	15.9±3.07	12.9±1.40	14.3±6.86	11.7±0.59			
Whole blood 5-HT T3	7.1±2.73	4.3±1.11	−	8.1±0.47		***	−
Platelet 5-HT uptake T3	59.1±7.98	35.7±3.35	−	40.3±1.53	*		−
Platelet 5-HT level T1	20.2±4.54	21.8±2.01	15.6±10.18	21.3±0.77			
Platelet 5-HT level T2	28.0±5.42	23.7±2.42	22.2±12.15	20.1±0.98			
Platelet 5-HT level T3	18.8±6.56	14.4±2.61	−	20.2±1.03		*	−
***Phase 3***	*n = 1–2*	*n = 42–51*	*n = 6*	*n = 158–170*			
Whole blood 5-HT T1	8.1±4.01	11.5±0.87	14.6±2.45	11.1±0.51			
Whole blood 5-HT T2	9.6±4.89	11.3±1.04	13.9±2.98	12.2±0.62			
Whole blood 5-HT T3	4.0±3.87^y^	6.3±0.88^yz^	11.0±2.42^z^	7.9±0.54^z^			*
Platelet 5-HT uptake T3	42.4±16.20	38.9±2.71	32.3±7.13	40.8±1.59			
Platelet 5-HT level T1	16.8±5.94	21.2±1.26	25.1±3.61	20.9±0.72			
Platelet 5-HT level T2	18.0±8.70	19.5±1.78	24.9±5.22	21.2±1.01			
Platelet 5-HT level T3	10.6±9.17^a^	15.9±2.00^ab^	29.2±5.63^b^	20.3±1.16^b^		*	**

a Untransformed LSmeans. LSmeans lacking a common letter differ by P<0.05 (a/b) or P<0.10 (y/z).

b Whole blood 5-HT level in nmol/ml; Platelet 5-HT level in nmol/platelet 10^9^; Platelet 5-HT uptake velocity in pmol/platelet 10^9^/min.

c B = Main effect of tail biter; V = Main effect of victim; B×V = Interaction between main effects resulting in Biter, Victim, Biter/Victim and Neutral.

−Not tested.

+P<0.10.

*P<0.05.

**P<0.01.

***P<0.001.

### Performance in behavioural tests by different types of pigs

#### Principal component analysis

In B housing, six factors were retained from the PCA (Eigenvalue or EV>1). However, only five factors were retained from the PCA to maintain an equal number of factors for both B and E housing as in E housing five factors had an EV>1 (Table 5 and 6). The factors of B housing together explained 87% of the total variance and of E housing this was 93%. Factors retained in B and E housing were largely similar. Each factor was labelled according to the importance of measures defined by loadings. The factor ‘Early life exploration’ had high positive loadings for the proportion of time exploring the novel arena and walking, and a high negative loading for the proportion of time spent standing alert during the pNEe. The proportion of time spent exploring the bucket presented during NOe loaded positively, and proportions of time spent near the wall zone and standing alert loaded negatively (although this was for the latter variable less prominent in E housing), on the factor ‘Near bucket. The factor ‘Cortisol’ summarized mainly basal cortisol and the area under the curve, and to a lesser extent also changes (i.e. recovery) in cortisol determined around the novelty test post-weaning. In B housed pigs, high scores on the factor ‘Vocalizations & standing alert’ were associated with high frequencies of vocalizing during pNEe and NOe and with a low proportion of time spent exploring the arena during NOe. The loading pattern of this factor was slightly different in E housed pigs: here the factor ‘Vocalizations and standing alert’ was less clearly determined by frequencies of vocalizations during pNEe and NOe (with moderate loadings of 0.20 and 0.41, respectively), but had a high positive loading of the proportion of time spent standing alert, and a high negative loading of the proportion of time spent exploring the arena during NOe. Variables recorded during the back test, i.e. numbers of struggles and vocalizations, exclusively loaded on the factor labelled ‘Back test activity’.

**Table 5:** Loadings^a^ on the first five factors extracted by principal component analysis, after orthogonal rotation, of variables recorded in barren housed pigs (n = 212) during a back test at 2 weeks of age, a novel environment test at 3 weeks of age and a novel object test at 13 weeks of age, including saliva cortisol variables.

Measures	Early lifeexploration	Nearbucket	Cortisol	Vocalizations &standing alert	Back testactivity
***Back test***					
No. of struggles	−0.02	−0.05	−0.02	0.14	***0.72***
No. of vocalizations	−0.05	−0.04	0.03	*0.34*	***0.71***
***Pre-weaning novel environment exposure***					
Exploration (%)	***0.76***	0.01	−0.05	−0.14	0.01
Walking (%)	***0.77***	0.03	0.01	0.18	0.04
Standing alert (%)	−***0.83***	−0.03	0.05	−0.19	−0.04
No. of vocalizations	0.22	0.03	−0.03	***0.65***	0.06
***Novel object exposure***					
No. of vocalizations	−0.07	0.02	−0.04	***0.63***	0.11
Standing alert (%)	−0.23	−***0.65***	0.02	0.20	−*0.45*
Exploration (%)	0.25	0.10	−0.08	−***0.50***	*0.49*
Exploring bucket (%)	−0.02	***0.81***	0.04	0.27	−0.10
Distance covered (m)	0.23	0.18	0.01	*0.47*	0.11
At door zone (%)	−0.04	−0.26	−0.04	0.07	0.03
At wall zone (%)	0.10	−***0.74***	−0.01	−*0.32*	0.11
***Salivary cortisol***					
Basal cortisol	−0.10	0.17	***0.83***	0.01	−0.13
Cortisol change (t = 15–t = 0)	0.08	−0.10	0.18	−0.06	0.23
Cortisol change (t = 15–t = 60)	0.04	−0.06	***0.52***	−0.03	0.17
Cortisol area under the curve	−0.09	0.11	***0.85***	0.01	−0.05
***Variance explained (%)***	**24.20**	**20.59**	**15.36**	**14.15**	**12.84**

Proportions of total variation explained by each factor are given.

a Loadings >0.30 or <−0.30 are indicated in italics, and loadings >0.50 or <−0.50 are also indicated in bold.

**Table 6:** Loadings^a^ on the first five factors extracted by principal component analysis, after orthogonal rotation, of variables recorded in enriched housed pigs (n = 212) during a back test at 2 weeks of age, a novel environment test at 3 weeks of age and a novel object test at 13 weeks of age, including saliva cortisol variables.

Measures	Early lifeexploration	Nearbucket	Cortisol	Vocalizations& standing alert	Back testactivity
***Back test***					
No. of struggles	−0.09	−0.07	0.08	−0.05	***0.72***
No. of vocalizations	−0.01	−0.04	0.12	−0.03	***0.80***
***Pre-weaning novel*** ***environment exposure***					
Exploration (%)	***0.75***	0.01	0.02	−0.08	−0.16
Walking (%)	***0.78***	0.02	0.03	−0.03	0.23
Standing alert (%)	−***0.78***	−0.03	−0.05	−0.01	−0.08
No. of vocalizations	0.24	0.05	−0.03	0.20	*0.42*
***Novel object exposure***					
No. of vocalizations	0.08	0.14	−0.16	*0.41*	*0.32*
Standing alert (%)	−0.19	−*0.33*	−0.01	***0.82***	−0.12
Exploration (%)	0.02	−*0.36*	0.05	−***0.82***	0.00
Exploring bucket (%)	0.07	***0.93***	−0.04	0.01	−0.02
Distance covered (m)	0.16	*0.36*	−0.19	−0.12	*0.41*
At door zone (%)	−0.01	−*0.33*	−0.01	−0.03	0.01
At wall zone (%)	−0.01	−***0.82***	0.09	−0.01	0.04
***Salivary cortisol***					
Basal cortisol	−0.01	−0.07	***0.85***	0.00	−0.02
Cortisol change (t = 15–t = 0)	−0.14	0.19	0.07	0.08	0.09
Cortisol change (t = 15–t = 60)	−0.06	0.02	−0.27	0.09	−0.05
Cortisol area under the curve	−0.04	0.06	***0.88***	0.04	−0.01
**Variance explained (%)**	**20.79**	**24.85**	**15.93**	**14.39**	**17.06**

Proportions of total variation explained by each factor are given.

a Loadings >0.30 or <−0.30 are indicated in italics, and loadings >0.50 or <−0.50 are also indicated in bold.

#### Barren housing

**Figure 2:**
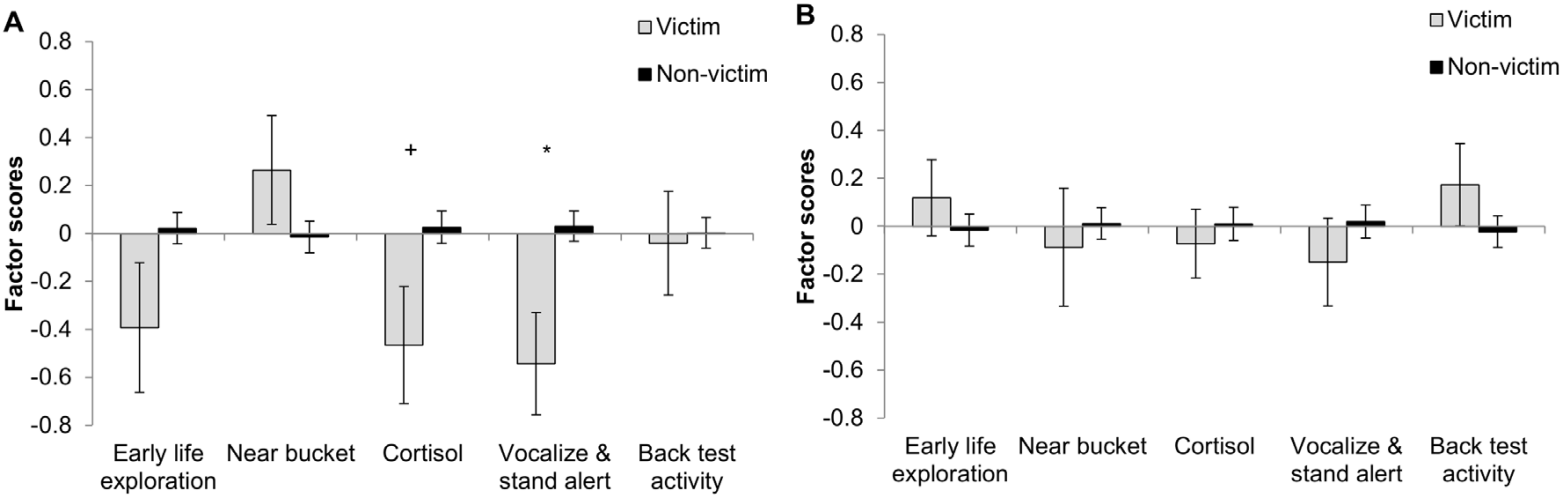
Factor scores of victims and non-victims of tail biting at time of weaning. Behavioural and physiological responses of pigs to novelty (pre- and post-weaning) were summarized in five factors using a PCA. Factor scores are presented per type of pig, i.e. victim (with tail wound) or non-victim (without tail wound) at time of weaning. **A:** Barren housing. **B:** Enriched housing. +P<0.10, *P<0.05.

**Figure 3:**
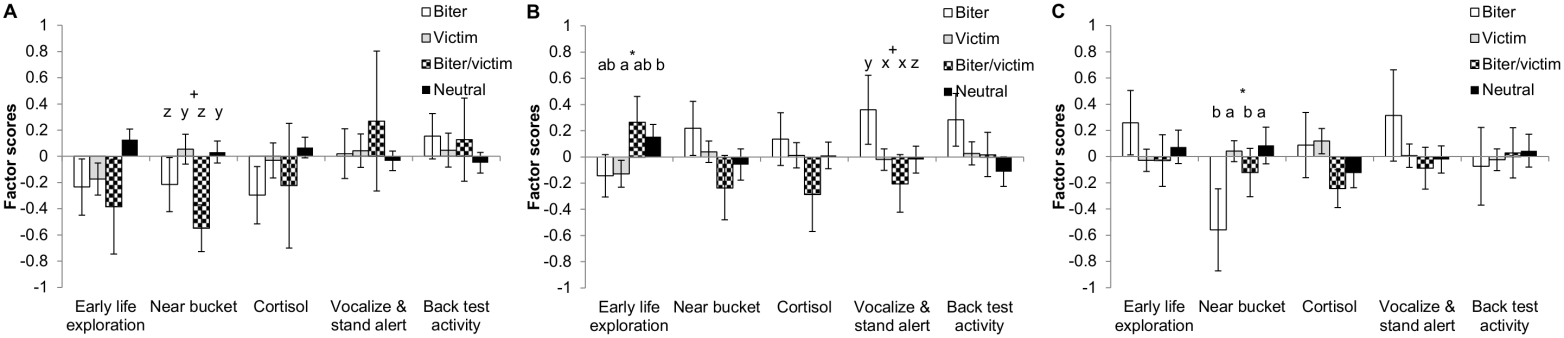
Factor scores of barren housed pigs classified as biters, victims, biter/victims or neutrals with respect to tail biting post-weaning. Behavioural and physiological responses of pigs to novelty (pre- and post-weaning) were summarized in five factors using a PCA. Factor scores are presented per type of pig, i.e. tail biter, victim (with tail wound), both tail biter and victim (biter/victim), or pigs that were neither tail biter or victim (neutral). **A:** Phase 1 post-weaning. **B:** Phase 2 post-weaning. **C:** Phase 3 post-weaning. +P<0.10, *P<0.05. Pig types lacking a common letter differ significantly (a/b) or tend to do so (x/y/z).

Overall, B housed pigs classified as tail biters had significantly lower ‘Near bucket’ scores compared to non-tail biters (tail biters: −0.24±0.14, non-tail biters: 0.12±0.11, P<0.05). Furthermore, tail biters tended to have a higher ‘Back test Activity’ compared to non-tail biters (tail biters: 0.21±0.13, non-tail biters: −0.09±0.11, P<0.10) and more specifically it tended to be so compared to victims and neutral pigs (biter: 0.42±0.24, victim: −0.02±0.09, neutrals: −0.17±0.19, P<0.10). No other significant (or tendencies to) relationships between the overall classification of pigs and PCA factors were found (data not shown). When considering phases of life separately, piglets with a tail wound at weaning had lower ‘Vocalizations & standing alert’ scores (P<0.05) and tended to have lower ‘Cortisol’ scores (P<0.10) (Figure 2A). Tail biters identified during phase 1 tended to have lower ‘Near bucket’ scores (P<0.10), and tail biters of phase 3 had significantly (P<0.05) lower ‘Near bucket’ scores compared to non-tail biters (Figure 3A and 3C). During phase 2 post-weaning, an interaction between ‘biter’ and ‘victim’ was found with respect to ‘Early life exploration’, where victims had significantly lower scores of this factor compared to neutral pigs (P<0.05), but not compared to tail biters and biter/victims (Figure 3B). Furthermore, victims identified during phase 2 tended to have lower ‘Vocalizations & standing alert’ scores compared to non-victims (P<0.10), but an interaction between ‘biter’ and ‘victim’ tended to be present as well and revealed that tail biters seemed to have higher scores compared to victims, biter/victims and neutrals (all P<0.10) (Figure 3B).

#### Enriched housing

**Figure 4:**
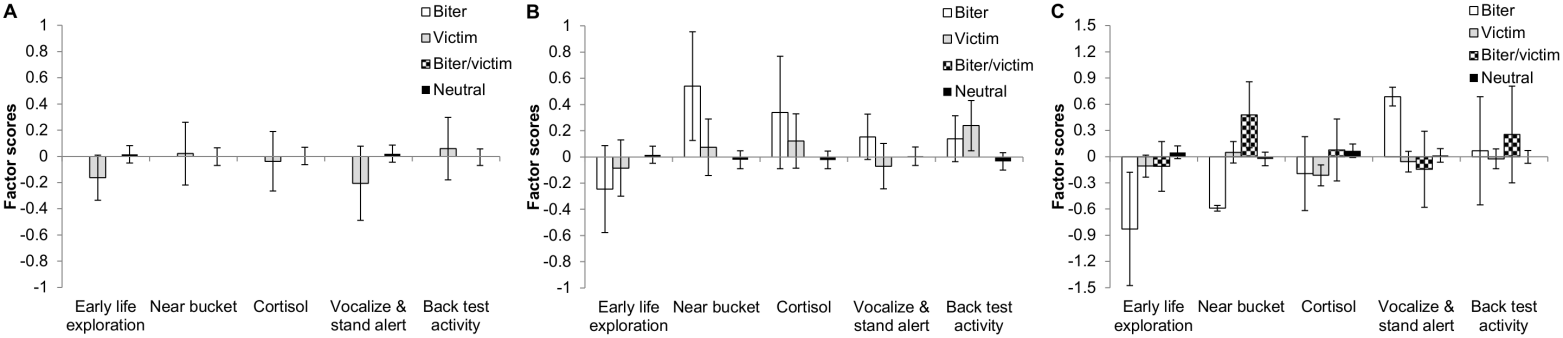
Factor scores of enriched housed pigs classified as biters, victims, biter/victims or neutrals with respect to tail biting post-weaning. Behavioural and physiological responses of pigs to novelty (pre- and post-weaning) were summarized in five factors using a PCA. Factor scores are presented per type of pig, i.e. tail biter, victim (with tail wound), both tail biter and victim (biter/victim), or pigs that were neither tail biter or victim (neutral). **A:** Phase 1 post-weaning. **B:** Phase 2 post-weaning. **C:** Phase 3 post-weaning.

Classifying the E housed pigs once (‘overall’) with respect to tail biting and thereby combining all phases of life, did not reveal any relationships with the factors obtained from the PCA (‘Early life exploration’, ‘Cortisol’, ‘Near bucket’, ‘Vocalizations & standing alert’, and Back test activity’) (data not shown). Neither having a tail wound at the time of weaning (Figure 2B) nor type of pig post-weaning had an effect on the five factors (Figure 4).

### Correlations between blood 5-HT and performance in behavioural tests

#### Barren housing

In B housed pigs, ‘Vocalizations & standing alert’ was significantly positively correlated with platelet 5-HT levels determined at T1 (r = 0.15, P<0.05) and it tended to be so for T2 (r = 0.13, P<0.10) and T3 (r = 0.12, P<0.10). Furthermore, also ‘Early life exploration’ tended to be positively correlated with platelet 5-HT levels determined at T1 (r = 0.12, P<0.10) and T2 (r = 0.12, P<0.10). No other correlations were found between the factors retained from the PCA and 5-HT measures.

#### Enriched housing

In E housed pigs, ‘Back test activity’ was significantly positively correlated with whole blood 5-HT measured at T1 (r = 0.23, P<0.001) and T2 (r = 0.20, P<0.01), and platelet 5-HT levels of T1 (r = 0.20, P<0.01) and T2 (r = 0.23, P<0.001). Furthermore, ‘Cortisol’ tended to be negatively correlated with whole blood 5-HT levels at T2 (r = −0.13, P<0.10) and with platelet 5-HT levels at T2 (r = −0.14, P<0.10).

## Discussion

The main aim of our study was to explore whether tail biting in pigs is associated with behavioural and physiological responses to challenges, and also with blood serotonin. To our knowledge, this experiment is the first to provide evidence suggesting that tail biting in pigs is possibly linked to both fearfulness and the blood 5-HT system.

### The effect of housing

Platelet serotonin (5-HT) uptake velocities were significantly higher in barren (B) compared to enriched (E) housed pigs. Except for one tendency for higher 5-HT platelet storage in B housed pigs than in E housed pigs, no housing effects on blood 5-HT levels were found. In a previous study in pigs [22], housing affected blood platelet 5-HT measures also only marginally and not significantly. Blood 5-HT has been associated with, amongst others stress physiology [45], [46], gut motility [47], and immune function [48] which may be affected by type of housing. B housed pigs are likely to suffer from stress due to the inability to perform species specific behaviours [49]. In humans, stress may reduce platelet 5-HT uptake sites (patients with PTSD [45]) and velocity (after surgery [46]). This altered platelet 5-HT uptake may be related to changes in HPA-axis responses, although cause and effect remain undecided [46]. Environmental enrichment in pigs, as compared with barren housing, has been shown to affect HPA-axis (re)activity at different levels indeed [50], [51] and also in our E and B pigs differences in cortisol levels (but not in response to novelty) were found (see [30]). Furthermore, in E housed pigs a tendency for a negative correlation between salivary cortisol and 5-HT storage was observed, suggesting a link between HPA-axis functioning and platelet 5-HT. In addition, E housed pigs likely have an increased gut motility [52] due to the availability of highly fibrous straw. In human babies, increased gut motility was related to increased platelet 5-HT levels [47], and in chickens the availability of fibrous litter for foraging, which likely affects gut motility, also affected levels of blood 5-HT [53]. Also in pigs, provision of dietary fibres has been shown to affect whole blood 5-HT [54]. Hence, also gut motility may relate to blood 5-HT (uptake) in different ways. Collectively, these findings suggest that the effect of housing on platelet 5-HT uptake velocity in our pigs may be related to both stress (B pigs) and the presence of fibrous foraging material, i.e. straw (E pigs). However, the exact underlying mechanisms remain unclear and the existence of other aspects involved cannot be excluded, as peripheral 5-HT also serves other functions and plays, for instance, a role in immune responses [48].

Relationships between behaviours may be affected by the environment (e.g. [55], [56]), which emphasizes for our study the importance of studying relationships between tail biting and other pig characteristics in different housing systems. Nevertheless, in E housing far less tail biting and tail damage was observed and only few E housed tail biters were identified [6]. Consequently, in E housed pigs, relations between tail biting and serotonergic, behavioural and cortisol measures were explored, but results should, given these low numbers of tail biters, be considered with caution.

### Tail biting and its relation with blood serotonin

Classifications of pigs according to performing and/or being the victim of tail biting at any phase of life, i.e. over the whole observation period, did not relate to peripheral serotonergic measures. Tail biting behaviour observed in individual pigs was, however, inconsistent throughout life [6], which may explain the lack of relationships between this ‘overall’ characterization with respect to being the actor and recipient of tail biting (combination of all phases in life) and 5-HT measures. However, our results also show that relationships between tail biting and 5-HT seemed to be present in pigs of both housing systems when specific phases in a pig’s life were considered. Generally, in several phases of life (although not all), tail biters had lower whole blood and platelet 5-HT levels and higher platelet 5-HT uptake velocities compared to either non-tail biters or other types of pigs (victims, biter/victims, neutrals). This seems in line with lower 5-HT values in whole blood or platelets found in humans with mental disorders such as obsessive compulsive disorders (OCD) [57] and depression [58], [59], and in laying hens that perform feather pecking behaviour [60], [61]. Additionally, in laying hens selected for a low mortality due to severe feather pecking and other injurious behaviours, lower platelet 5-HT uptake velocities were found [60], [62]. Therefore, tail biters may suffer from a (temporary) change in the blood 5-HT system. Remarkably though, victims of tail biting (i.e. pigs with a tail wound) also seemed to have lower whole blood and platelet 5-HT levels, but had a significantly lower or tendency for a higher platelet 5-HT uptake velocity, compared to non-victims. It must be noted, though, that non-victims were possibly also tail bitten and could have bite marks, but they were not as severely bitten as the pigs we identified as victims. It is possible, that victims with tail wounds may suffer from depressive-like symptoms (e.g. due to B housing, to being tail bitten, or a combination of both) and also develop a malfunctional blood 5-HT system, although not entirely identical to that observed in tail biters. In pigs, especially the neutral animals not involved in the damaging behaviour at all, were found to differ in gene expression profiles (some of the genes were associated with production, sociality, and novelty seeking) compared to animals that either displayed or received the damaging behaviour [27]. Although in our study both tail biters and victims were found to differ from non-tail biters or non-victims, or other pig types, a clear and consistent difference between neutral pigs and both tail biters and victims was not found. This difference in findings may be explained by the type of pigs used and the relatively low tail biting threshold (>1 tail biting incidence per phase of life) that was used. In the current study, we used all available animals including a group of pigs that were both tail biter and tail bite victim, instead of a selected set of (possibly the most extreme) animals (biter, victim, neutral) as was done by others [27].

In B housed pigs, whole blood and platelet 5-HT measured at T2 (i.e. week 9) were related to tail biting in phases 2 and 3 post-weaning. In E housing, associations between type of pig, with respect to tail biting and 5-HT measures were found at T3 (i.e. week 22) only. Additionally, 5-HT measures determined at T1 (i.e. week 8) were not significantly related to any type of pig in any phase, except for pigs that were victims of tail biting at time of weaning and subsequently B housed. It seems, therefore, plausible that, rather than suffering from a malfunctional blood 5-HT system from early life onward, pigs of both housing systems develop changes in their blood 5-HT system during life. Average blood 5-HT values determined at T1 and T2 were similar, most likely because the time span between the two samples was very short and decreases in 5-HT levels due to aging, as found in humans [63], were not yet present. However, significant associations between tail biting and blood 5-HT were present in B housed pigs at T2. This suggests that temporarily mixing unfamiliar pigs can trigger relationships between tail biting and blood 5-HT when also unfavourably housed. As our blood samples were taken 3 days after mixing the pigs and the half life time of 5-HT in blood is approximately 3–4 days [47], [64], it is not impossible that some changes in the blood 5-HT system of individual pigs developed after mixing. Especially when mixing resulted in a lower feed intake [65] and consequently limited dietary tryptophan intake that is needed to synthesize 5-HT [66]. Dietary tryptophan levels [67] and blood tryptophan measures have been associated with tail biting behaviour in pigs [68]. Additionally, correlations between blood 5-HT values determined at different time points were not extremely high (although clearly and significantly present), suggesting fluctuations in blood 5-HT levels of individual pigs. Remarkably, in E housing also some relations were found between type of pig and 5-HT measures in later life. Here, changes in the 5-HT system may have developed due to a change in tryptophan availability or demand caused by a depressed feed intake, stress [69] (e.g. due to restricted space [70] caused by a pig’s growth) or gut (mal)functioning [47], [71], although this may also be true in B housed pigs. According to our results, pigs involved in tail biting do not consistently exhibit changes in the blood 5-HT system that may have led to tail biting behaviour or becoming a victim of tail biting. However, in phases of life during which individual pigs display tail biting, a relationship with the 5-HT system seems to exist, suggesting that tail biting with its accompanying blood 5-HT levels (lower 5-HT storage and higher platelet uptake velocity in tail biters) fluctuate throughout time. The present findings seem to support the existence of a relationship between the blood 5-HT system and tail biting [68], similar to the relationship between 5-HT and feather pecking in laying hens [60], [61], but in our pigs this relationship seems more state-like than trait-like. This does, however, not exclude the possible existence of trait-like differences in 5-HT system functioning in other types of tail biters (such as obsessive tail biters, see also [2]) than observed in our study (likely two-stage tail biters, see [6]), which may predispose these animals to perform tail biting behaviour.

### Tail biting and its relation with responsiveness in pigs and blood serotonin

A fairly consistent relationship was observed in B housed tail biters with the factor ‘Near bucket’ (retained from a PCA) during a novel object test post-weaning, i.e. tail biting pigs spent or tended to spend less time near a metal bucket introduced during a novel environment test, and spent more time standing alert (a vigilance behaviour [72]), and in the wall zones compared to non-tail biters. In E housing no such relation was found, probably due to the low level of tail biting observed. Avoiding a novel object [73], standing alert [74], [75], and seeking the walls of an arena (also called ‘Thigmotaxis’) [76], [77] altogether suggests the presence of a higher fearfulness in B housed tail biters at times of challenge in an unfamiliar environment and without other pigs present. Also in humans with behavioural disorders [78], [79] or other animals displaying maladaptive behaviour (e.g. poultry: [19], [80], dogs: [81], and cats: [82]) higher levels of fear or anxiety were suggested. Furthermore, the lower blood 5-HT levels in our tail biters in combination with a higher fearfulness seem to support previous findings in pigs where lower levels of exploring an unfamiliar arena (indicative of higher fearfulness [83]) was associated with lower whole blood and platelet 5-HT levels [22]. However, in a different study, it was suggested that tail biting pigs were less fearful compared to victim pigs, which was based on the shorter latency time to touch a novel object in the home pen, the longer contact duration with the novel object and lower levels of locomotion [20]. Notably, however, in this particular study tail biters responded to exposure to the novel object test with a decrease of heart rate variability in comparison with victims; this indicates suppression of the parasympathetic nervous system which is also found in human patients with panic disorders [84]. Moreover, different measures of heart rate variability were found to be significantly intercorrelated in tail biters but not in victims or control pigs [20]; similar intercorrelations were demonstrated in human panickers [84]. The present finding in tail biting pigs, therefore, seem to agree with the notion that altered 5-HT system functioning, high fearfulness and high levels of impulsive behaviours are related (see [85] for a review).

Over all phases (but not in separate phases), being a tail biter in our study tended to be associated with a higher ‘Back test activity’ (indicative of a proactive coping strategy [16], [24]), compared to a non-tail biter. This is in line with previous findings in parrots [86], where feather pickers likely had a proactive coping strategy. In laying hens it was suggested that the initial (first order) feather peckers may be proactive copers [87], whereas the hens attracted to already damaged or ruffled feathers (second order peckers) may be reactive copers [12]. Interestingly, in one life phase, tail biters also tended to have higher ‘Vocalizations & standing alert’ scores compared to victims, biter/victims and neutrals, whereas victims had lower scores of this factor compared to non-victims. Moreover, B housed pigs with higher ‘Vocalizations & standing alert’ scores had higher platelet 5-HT levels, and E housed pigs with a higher ‘Back test activity’ also had higher whole blood and platelet 5-HT levels. Altogether, this suggests that blood 5-HT measures in pigs may be related to more than one personality dimension, which was also proposed by others [85]. Although the relationship between tail biting and coping strategy was not significantly nor consistently present, considering interactions between 5-HT, fearfulness and other personality dimensions may be relevant in understanding problematic behaviours such as tail biting in pigs.

## Conclusions

Generally, within specific phases of life, tail biters and to a lesser extent also victims seemed to have lower levels of blood serotonin compared to non-performers/receivers. Tail biters also seemed to have higher blood platelet uptake velocities. Furthermore, our results show the importance of considering different phases in a pig’s life with respect to relationships between (problematic) behaviours and serotonergic measures as both may fluctuate in time. Additionally, barren housed tail biters seemed more fearful after a challenging event. Taken together, considering both behavioural responses to challenging events and blood serotonergic measures in pigs may help in characterizing and identifying individuals at risk for developing damaging behaviours such as tail biting. Further research is needed to elucidate the nature of the relationship between peripheral 5-HT and tail biting, and to develop successful preventive strategies and interventions.
